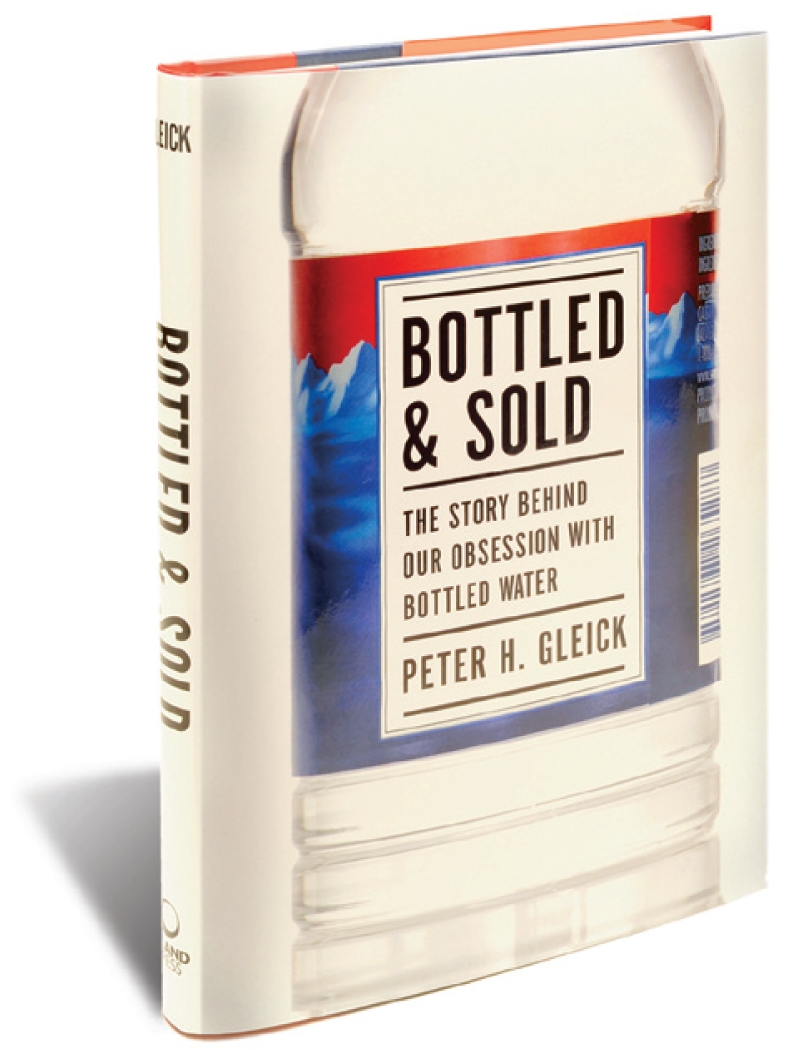# Bottled & Sold: The Story Behind Our Obsession with Bottled Water

**Published:** 2011-05

**Authors:** David Beckman

**Affiliations:** David Beckman directs the Water Program at the Natural Resources Defense Council, one of the nation’s largest environmental advocacy organizations. The Water Program focuses on assuring safe and sufficient water for people and the environment in the United States. He is graduate of the University of California, Berkeley, and Harvard Law School

In *Bottled & Sold,* Peter Gleick offers a broad indictment of the bottled water industry, which Gleick asserts has successfully pulled the wool over the eyes of a generation of Americans who would be better off drinking from the tap. Criticism of bottled water is not new; for more than a decade, environmental organizations and others have observed that the water in the bottle is often of no better quality—and sometimes may be worse—than tap water. But Gleick is not content to address only the dubious qualitative claims that bottled water is better water. Instead, the Pacific Institute President and MacArthur Foundation “genius” award winner serves up a wide-ranging screed, taking aim at the hucksters peddling miracle water, the international bottling companies sucking up so much water that they are altering ecosystems, and a scattershot regulatory system that offers only modest protection to consumers.

If you are familiar with Gleick’s important research on water, the tone may surprise you. If a lot of his work is tightly constructed—more like a classical musical composition—then *Bottled & Sold* is, by contrast, a jazz riff. Gleick weaves together some original research with offerings from a wide range of analysts and authors, and plays all of this off his own observations gleaned from travels to bottling plants and industry conventions. Gleick covers the waterfront, chronicling the public’s (often irrational) fear of tap water; the distinctions between the U.S. Environmental Protection Agency’s regulation of public water supply and the Food and Drug Administration’s less comprehensive oversight of the bottled product; lax labeling; and the range of social costs, from energy to environment, associated with bottled water. He saves his harshest critique for the hucksters on the fringe of the industry offering product like “rhythm-structured water,” who to Gleick are nothing more than modern snake-oil salesmen. Indeed, he devotes a significant portion of this volume to these characters, and these pages are particularly entertaining. But it might have been more illuminating to dive deeper on a more central question: how less colorful but more powerful *mainstream* bottlers successfully sell for a few dollars a product that most Americans can drink at home nearly free.

Indeed, Gleick’s most fundamental point is not fully apparent until the concluding chapter, in which the author suggests thoughtfully that we are entering a “Third Water Age” (which Gleick contrasts to the “Second Water Age,” when humans achieved the ability to alter and manage the hydrological cycle, and did so with great public works projects such as aqueducts and damns). To Gleick, water 3.0 is about the “soft path,” which he characterizes as “a more sustainable approach that recognizes the realities of a renewable but ultimately limited resource.” Bottled water, in Gleick’s view, is a symptom of the ultimate failure and inadequacy of the “hard path” followed during the Second Water Age—the privatization, if you will, of what was and should be a universally available public resource.

However sympathetic a reader may be to this suggestion, it is hard to follow Gleick there without reservation. If, as Gleick asserts, tap water is often as tasty and clean, and maybe tastier and cleaner, than bottled water, then it is not the failure of the 19th- and 20th-century systems that deliver water to the public that is the problem (the Second Water Age), it is the disjunction between fact and perception that is distorting water policy. And while he certainly acknowledges it, Gleick perhaps still underplays America’s love of pure convenience in the rise of bottled water. Indeed, Americans do not just want their water in an easy to take-along container. We increasingly want—and expect—*everything* to be portable: our music, food, information, communications, and entertainment, too.

Whether or not bottled water is a harbinger of the need for a new, third approach to water or not, Gleick is clearly correct in leading the charge for that new course. Water scarcity—which in many places in the country is a fact, and in many more will become one in the decades to come—is ignored by Americans at our own peril. Water pollution, a leading cause of which is, quite literally, all of the concrete in urban America which prevents natural water recharge of aquifers and generates polluted runoff, will not be entirely tamed without the greener, softer path Gleick discusses. Even if bottled water did not become a consumer powerhouse solely because of systemic failures in how we approach water, it clearly is a powerful metaphor of those failures. Metaphors aside, *Bottled & Sold* ultimately is a satisfying and enjoyable read, one that offers important information and raises questions that will only grow more critical to answer in the years and decades to come.

## Figures and Tables

**Figure:**